# RNA viruses in trypanosomatid parasites: a historical overview

**DOI:** 10.1590/0074-02760170487

**Published:** 2018-02-19

**Authors:** Danyil Grybchuk, Alexei Y Kostygov, Diego H Macedo, Claudia M d’Avila-Levy, Vyacheslav Yurchenko

**Affiliations:** 1 University of Ostrava, Faculty of Science, Life Science Research Centre, Ostrava, Czech Republic; 2 Fundação Oswaldo Cruz-Fiocruz, Instituto Oswaldo Cruz, Laboratório de Estudos Integrados em Protozoologia, Coleção de Protozoários, Rio de Janeiro, RJ, Brasil

**Keywords:** trypanosomatidae, RNA viruses, leishmaniavirus

## Abstract

Viruses of trypanosomatids are now being extensively studied because of their diversity and the roles they play in flagellates’ biology. Among the most prominent examples are leishmaniaviruses implicated in pathogenesis of *Leishmania* parasites. Here, we present a historical overview of this field, starting with early reports of virus-like particles on electron microphotographs, and culminating in detailed molecular descriptions of viruses obtained using modern next generation sequencing-based techniques. Because of their diversity, different life cycle strategies and host specificity, we believe that trypanosomatids are a fertile ground for further explorations to better understand viral evolution, routes of transitions, and molecular mechanisms of adaptation to different hosts.

Viruses are obligate intracellular parasites having no metabolism on their own. Instead, they employ resources from their hosts. Because of their high adaptability and diversity, viruses are the most abundant biological objects on Earth (Forterre 2010). Indeed, they can parasitise all cellular types; cases of simultaneous co-infection by different viral species are widespread. This observation indicates a deep evolutionary connection between cells and viruses possibly dating back to the origin of life itself (Forterre & Prangishvili 2009, Moreira & López-García 2009). Understandably, the virus research is mostly focused on the disease-causing agents of humans and livestock. However, the rise of methods for massively parallel nucleic acid sequencing enabled broad-scale studies of viral ecology and diversity in those groups of hosts, which were previously neglected (Cook et al. 2013, Li et al. 2015, Shi et al. 2016 , Aswad & Katzourakis 2017). In particular, this lead to the significant progress in the study of viruses in protists (La Scola et al. 2003, Blanc et al. 2015, Maumus & Blanc 2016, Schulz et al. 2017). One of the better studied groups in this respect are trypanosomatids, flagellate parasites of vertebrates, including humans and domestic animals, plants and invertebrates (Maslov et al. 2013, Lukeš et al. 2014).

*Methods of RNA viruses’ detection in parasitic protists* - Early reports of virus-like particle (VLPs) in various protists were mostly based on transmission electron microscopy (TEM), which was a part of routine species description procedures since 1950s (Anderson 1959). Unfortunately, for the most part, microscopic images were inconclusive and insufficient to prove the viral infection (Patterson 1990, Wang & Wang 1991). Moreover, nucleic acids sequencing methods were not well developed at that time further complicating description of new viruses from protists. Standards of virology require enrichment of viral particles by ultracentrifugation with subsequent demonstration of their infectivity in the original host (Hamilton et al. 1981). This technique had limited success with viruses of protists, since usually they cause only latent infections (Miller et al. 1988a, b). Not all of the early descriptions of putative protist-infecting viruses withstood further scrutiny. For example, nuclear filamentous and cytoplasmic polyhedral VLPs from *Entamoeba histolytica* were extensively studied by TEM throughout 1970s (Diamond et al. 1972, Mattern et al. 1972, 19, 1977). However, all attempts to purify VLPs failed and the nature of supposed viral agent has not been revealed thus far (Hruska et al. 1973, Banik et al. 2014).

Another, more fruitful approach of studying viruses in protists is based on observation of double-stranded RNA (dsRNA) in cellular RNA extracts. This method facilitated discovery of dsRNA viruses, but was also successfully applied for single-stranded RNA (ssRNA) viruses, which produce small amounts of dsRNA replication intermediates (Cai et al. 2009). Originally, it was based on ribonuclease A treatment of DNA-free RNA samples at various salt concentrations, as this enzyme has lower affinity toward dsRNA at higher ionic strength of the reaction buffer (Wang & Wang 1986). Other methods of dsRNA isolation included salting-out ssRNA molecules with high concentration of LiCl (Potgieter et al. 2009) or digestion with single-stranded-specific S1 nuclease (Vogt 1973). Regardless of the exact method used, viral dsRNA was visualised in the agarose gel and used for downstream applications (sequencing or Northern blot) (Tarr et al. 1988, Scheffter et al. 1994). These methods allowed viral discoveries in *Trichomonas*, *Giardia*, and *Leishmania* spp. (Wang & Wang 1986a, b, Tarr et al. 1988). Also, virus particles were isolated form these cells using CsCl gradient ultracentrifugation, and association of dsRNA with these particles was confirmed (Miller et al. 1988, Widmer et al. 1989, Khoshnan & Alderete 1993). A recently developed real time quantitative polymerase chain reaction (RT-qPCR)/ultracentrifugation-based protocol for viral purification couples molecular identification with TEM and has a potential to greatly facilitate viral detection (de Souza et al. 2014).

The rate of new RNA virus discovery accelerated with the advent of high-throughput sequencing analyses of environmental samples (Kristensen et al. 2010, Mokili et al. 2012). The dawn of next-generation sequencing era coincided with a gradual shift of attention from human-pathogenic viruses toward the broader study of associated commensal viruses from various biotopes (Dutilh et al. 2017). Although awe-inspiring, all these results must be taken with a caution because metatranscriptomic studies have a number of fundamental limitations. The most significant one is that it is not possible to unambiguously identify a true host of the virus. When a metatranscriptome of a macroscopic organism is concerned, there is a chance that the actual viral host is a unicellular symbiont or a parasite. Another vulnerability of such an approach is its reliance on homology search algorithms to identify putative viral genes.

*Exploration of viruses in Trypanosomatidae by electron microscopy* - During second half of the last century there were numerous reports of VLPs in dixenous (= two hosts in the life cycle) trypanosomatids of the genera *Leishmania*, *Trypanosoma*, and *Phytomonas*. Historically, the very first observation of VLPs in Trypanosomatidae were made by Molyneux in *Leishmania hertigi* [now *Paraleishmania hertigi* (Kostygov & Yurchenko 2017)] isolated from the prehensile-tailed porcupine (Molyneux 1974). Groups of well discernable rounded electron-dense objects 55-60 nm in diameter were documented in the cytoplasm near kinetoplast. VLPs had regular arrangement (paracrystalline array) and were reported to segregate between dividing cells. Further ultrastructural studies demonstrated that VLPs had electron-dense outer ring and electron-translucent core and were sometimes associated with tubular cisternae and vesicular bodies. It was proposed that these unusual cytoplasmic structures might be manifestations of viral infection and could be implicated in VLP morphogenesis. However, continuous three-year long observations of *in vitro* cultured parasites revealed that VLPs were stably present and did not affect the cells (Croft & Molyneux 1979). In the same study *P. hertigi* promastigotes were differentiated in the mouse peritoneal macrophage model. There were less VLPs in the resultant amastigotes compared to their almost ubiquitous presence in promastigotes. Thus, it was concluded that the putative virus might go through a cryptic stage during trypanosomatid differentiation. Numerous attempts to purify virus particles and their nucleic acid from *P. hertigi* cultures failed. These negative results, together with disappearance of VLPs in amastigotes, casted doubts on their viral nature (Eley et al. 1987). Similar arrays of 40-80 nm particles of various shapes were also reported in four out of seven isolates of *Endotrypanum* spp. from sloths and *Lutzomyia* flies in Central America (Croft et al. 1980). However, the pleomorphy of particles and apparent absence of cytopathic effects pushed authors to assume their non-viral nature. The VLPs were also reported in *Trypanosoma melophagium* - a parasite of sheep transmitted by the ectoparasitic sheep ked (Molyneux & Heywood 1984), but were never characterised by modern molecular methods. Most recently, VLPs were documented in *T. cruzi*, an etiological agent of Chagas disease. The study describes two types of 32 and 48 nm particles located in endoplasmic reticulum, Golgi apparatus and cytoplasm of epimastigotes. The incidence of VLPs was only 2% and none were found in other life cycle stages. Apart from TEM, no additional analyses were performed to characterize a putative virus (Fernández-Presas et al. 2017).

The first monoxenous (= with one host) trypanosomatid, where VLPs were recorded, was the endosymbiont-bearing *Angomonas* (formerly *Crithidia*) *desouzai*. Similarly to the case of *P. hertigi*, VLPs were located in hexagonally organized groups near the nucleus and were observed in cells throughout 2 years of *in vitro* cultivation with no perceivable impact on the host (Motta et al. 1991). Further work showed that putative viral particles contained RNA, but not DNA (Soares et al. 1989, Motta et al. 2003), yet all attempts to isolate this RNA from *Angomonas* cultures failed. Viral particles and RNA were also absent in the recently described relative of *Angomonas*, *Kentomonas sorsogonicus* (Votýpka et al. 2014). Hence, presence of RNA virus in *A. desouzai* needs to be further validated.

Recently, VLPs were also documented in the monoxenous trypanosomatids *Leptomonas moramango* and *Crithidia pragensis*. In *L. moramango* clusters of round shaped electron-dense objects of around 55-60 nm were observed in proximity of kinetoplast and basal body, whereas in *C. pragensis*, the 35-40 nm VLPs were found inside the mitochondrion lumen (Yurchenko et al. 2014).

*Exploration of viruses in Trypanosomatidae by molecular methods* - In the early 1990s dsRNA, viruses of the family *Totiviridae* were discovered in different *Leishmania* spp. (Tarr et al. 1988, Widmer et al. 1989, Guilbride et al. 1992). *Leishmania RNA virus 1* (LRV1) from *L. guyanensis* M4147 was the first virus from kinetoplastids fully characterized in molecular terms. It was originally discovered as an unusual electrophoretic band of total nucleic acid extract. Further ultracentrifugation with subsequent negative-stain TEM revealed that RNA was associated with spherical proteinaceous particles 32 nm in diameter (Tarr et al. 1988). Partial sequence similarities between LRV1 and vesicular stomatitis virus (VSV) polymerase indicated that these viruses might be related. This view was supported by the fact that both VSV and *Leishmania* are carried by sand flies implying possible virus transfer between arthropods and trypanosomatids. Only four years later, when the sequencing of the full LRV1 genome was completed (Stuart et al. 1992), it became apparent that the two viruses are not related. Instead, the 3’-proximal ORF (ORF3) of LRV1 was shown to encode RNA-dependent RNA polymerase (RDRP) most similar to that of *Saccharomyces cerevisiae* L-A, a dsRNA virus of yeast. Similar viruses were described from other isolates of *L. guyanensis*, as well as from one isolate of *L. braziliensis* (Widmer et al. 1989, Guilbride et al. 1992). All these viruses from New World *Leishmania* originating from Amazon basin were assigned to the genus *Leishmaniavirus* within the family *Totiviridae* (Patterson & Larsen 1992) (Fig. 1). Totiviruses are known to infect a wide range of hosts, including protists (Hartley et al. 2012), fungi, yeast (Ghabrial et al. 2015), arthropods (Poulos et al. 2006), and vertebrates (Løvoll et al. 2010). All of them have at least two core proteins - capsid and RDRP (Fig. 2). In LRV1, the RDRP was identified as a product of ORF3 (Stuart et al. 1992). The upstream ORF2 encodes a capsid protein, as was demonstrated a few years later (Cadd & Patterson 1994a, b). These two ORFs overlap in all LRV1 representatives with no clear independent start codon for ORF3, leading to the conclusion that RDRP is translated only as a C-terminal extension of the capsid (Fig. 2). The fusion protein is thought to be produced by +1 ribosomal frameshift facilitated by stem-loop structures and pseudoknots within the overlap region (Scheffter et al. 1994). In mid-1990s research on leishmaniaviruses continued with the emphasis on various aspects of its molecular biology, such as virus-host interactions (Armstrong et al. 1993, Ro et al. 1997), mutational analysis of the capsid protein (Cadd et al. 1994a, b), and the mechanisms of translation and +1 ribosomal frameshift (Maga et al. 1995, Lee et al. 1996).

**Fig. 1:**
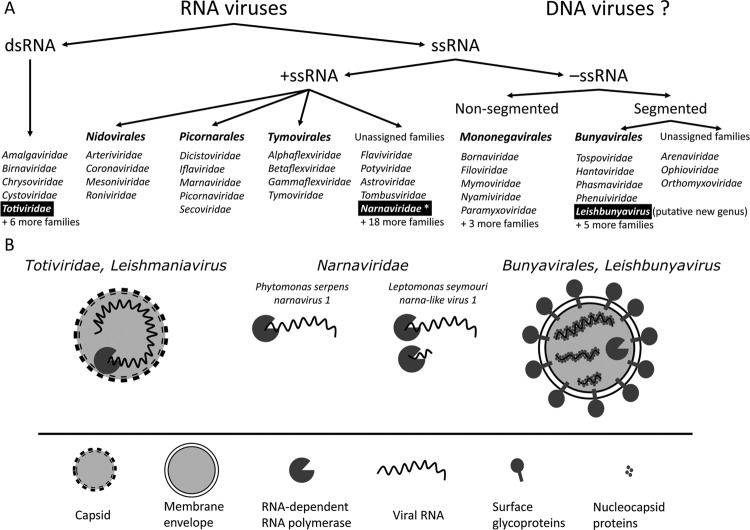
: described viruses of trypanosomatid in the context of RNA viruses’ diversity. Characterised representatives are highlighted and schematically depicted below the taxonomical summary. Asterisk marks the phylogenetic proximity, not the actual affiliation with the family.

**Fig. 2:**
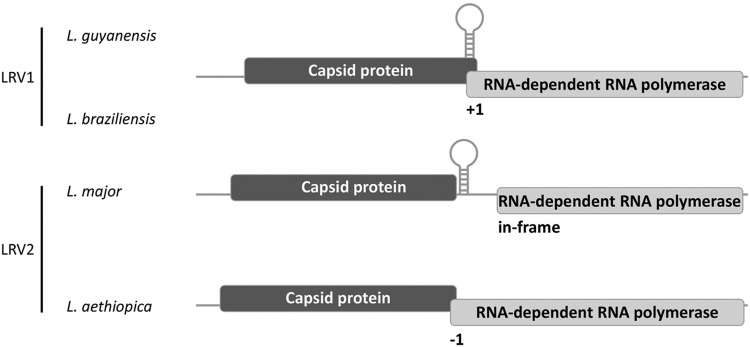
: genome organisation of LRV1/2 form various *Leishmania* spp. All leishmaniaviruses have two ORFs coding for capsid protein and RNA-dependent RNA polymerase (RDRP). The overlaps, putative secondary structures between the ORFs, and the reading frame of RDRP relative to capsid are indicated.

LRV1 is restricted to South America, yet its distribution is segregated between Amazon (North) and South, where *L. braziliensis* strains from Minas Gerais (Macedo et al. 2016) and Rio de Janeiro (Pereira et al. 2013) are not infected with the virus.

In 1995 the complete sequence of the new totivirus from the Old World *L. major* was reported (Scheffter et al. 1995). The virus was clearly related to LRV1, however, it was quite divergent in terms of amino acid sequences (only 38% and 47% identical amino acids in the capsid and RDRP proteins, respectively). Poor sequence conservation, probably, accounted for the failed detection by cross-reacting polyclonal antibodies raised against LRV1 capsid (Cadd et al. 1993). It also lacked the overlap between ORF2 and ORF3, suggesting either different mechanism of fusion protein translation or independent initiation of RDRP synthesis (Scheffter et al. 1995). The virus from *L. major* was assigned to the genus *Leismaniavirus* as LRV2 (Fig. 2, Supplementary data, Table).

Because of the differences in sequences and genome organizations of LRV1 and LRV2 (Fig. 2), it was proposed that these viruses diverged upon separation of Old and New World leishmanias. In support of this view, a study focused on virus-host co-evolution demonstrated similar topologies of phylogenetic trees inferred for virus-infected *Leishmania* isolates (6 American and 1 Asian, Supplementary data, Table) and respective viruses (Widmer & Dooley 1995). This model was in full agreement with the predominantly vertical transmission of totiviruses in yeasts (Fujimura & Esteban 2011) and asexual reproduction of *Leishmania* (Panton et al. 1991, Tibayrenc & Ayala 1991, Rougeron et al. 2017). Later, leishmaniaviruses were described in *L. aethiopica* and *L. infantum* isolates (Fig. 2, Supplementary data, Table), allowing even broader phylogenetic analysis (Zangger et al. 2014, Hajjaran et al. 2016). Taken together these results support the model of segregation of viruses to the Old and New World groups, further reinforcing the co-evolution hypothesis and posing a question on the role of virus in *Leishmania* infectivity or transmission.

The apparent evolutionary cause of virus retention was discovered only 15 years later, when it was demonstrated that LRV1 [strain LRV1-LgyM4147 (Adams et al. 2014)] interferes with vertebrate host immune response against *L. guyanensis* (Ives et al. 2011). This is the most studied model for LRV1 function. Viral dsRNA stimulates the production of pro-inflammatory interferon-β through the interaction with endosomal Toll-like receptor 3. This, in turn, tips the balance toward T-helper 1 mediated immune response leading to chronic inflammation and increased metastatic potential of *L. guyanensis* (Hartley et al. 2012, 2014). This results in enhanced dissemination and parasite persistence, which ultimately increases the chances of both *Leishmania* and its virus to be picked up by a sand fly and successfully complete their life-cycles (Márquez & Roossinck 2012). Thus, virus bearing presents a clear survival advantage in dixenous trypanosomatids. Another interesting question concerns RNA interference (RNAi). At first, it was thought that retention of dsRNA leishmaniaviruses is incompatible with RNAi, as the latter would counteract the selective advantage presented by virus-bearing (Lye et al. 2010). However, it was demonstrated that RNAi can be used to eliminate virus from the infected New World *Leishmania* by introducing small hairpin RNAs against viral genome. This implies that virus and RNAi are not mutually exclusive but instead are able to coexist in dynamical equilibrium (Brettmann et al. 2016). In addition, recent broad phylogenetic study of the distribution of RNAi components across Trypanosomatidae tree suggested at least seven independent losses of RNAi machinery in different trypanosomatid lineages and apparent lack of selective forces driving this process (Matveyev et al. 2017).

During the survey of trypanosomatid parasites isolates from lactiferous plants of the families *Euphorbiaceae* and *Apocynaceae*, *Phytomonas* spp. were reported to bear 34 nm virus particles containing 4.7 kb linear dsRNA (Marche et al. 1993). The virus presence was documented only in flagellates infecting coconut and oil palms, but not in trypanosomatids from other lactiferous plants growing in the same locality. The dsRNA genome, virion size and association with disease-causing unicellular parasite in many ways paralleled the situation with previously described totiviruses of *Leishmania*, *Trichomonas*, and *Giardia* spp. Nevertheless, absence of sequence data did not allow establishing the true identity of the virus at that time. Recently, a complete sequence of the virus infecting *P. serpens* isolated from the tomato fruit sap has been reported (Akopyants et al. 2016a, b). This virus appears to be a typical narnavirus, consisting of bare RNA molecule encoding RDRP (Fig. 1). Its closest relative is a narnavirus from the oomycete plant pathogen *Phytophthora infestans* (PiRV-4). Narnaviruses do not have capsids and possess only one positive sense single stranded genomic RNA almost entirely occupied by the RDRP gene. Much like totiviruses, they replicate in the cytoplasm and are transmitted either vertically or through cytoplasmic bridges between cells at the instance of mating (Ghabrial et al. 2015). Both *Phytophthora* and *Phytomonas* spp. are known to infect tomato, thus, a possibility of virus exchange between the two cannot be formally excluded.

The dsRNAs of viral origin were reported in *Leptomonas seymouri* using nuclease digestion assays and anti-dsRNA antibodies (Kraeva et al. 2015). Soon after, the sequences of viruses infecting *L. moramango* and *L. seymouri* were published (Akopyants et al. 2016b, Lye et al. 2016). The RDRP of *L. seymouri* virus had the highest sequence similarity to that of the narnavirus of *P. serpens*. However, it differed significantly from the latter by having additional smaller RNA segment with two putative ORFs with no recognizable homologs in the NCBI database (Lye et al. 2016) The function and nature of these segments remain to be investigated further. A recent study also reported high prevalence of *L. seymouri* narna-like virus in clinical isolates from visceral leishmaniasis patients co-infected with *L. donovani* and *L. seymouri*. Interestingly, the presence of virus and *L*. *seymouri* always correlated, suggesting that *L*. *donovani* cannot be the virus host (Sukla et al. 2017). Taken together with the observation that *L*. *seymouri* contains high amount of viral dsRNA (Kraeva et al. 2015), it is plausible to suggest that this narnavirus may modulate the pathogenicity of *L*. *donovani* similarly to the way the LRV does.

The negative sense ssRNA virus from *L. moramango* has a segmented genome consisting of large (L), medium (M), and small (S) fragments. Each segment was predicted to encode a single ORF. Of these, the L segment-encoded RDRP and the S segment-encoded nucleocapsid protein displayed notable homology to *Bunyaviridae* proteins. This virus was classified as a member of a new genus (tentatively, “Leishbunyavirus”) within the family *Bunyaviridae* (Akopyants et al. 2016a, b).

*Conclusions and perspectives* - The research on viruses in trypanosomatids has started in early 1970s and the early investigations were primarily driven by TEM. Development of molecular biology techniques led to the new discoveries, notably of leishmaniaviruses, which are now being extensively studied due to the role they play in *Leishmania* pathogenesis. Today, next generation sequencing techniques are greatly accelerating the pace of studies into viral diversity. Trypanosomatids are a fertile ground for further explorations. Indeed, there are numerous groups of these flagellates parasitizing a wide range of hosts (from invertebrates to plants and vertebrates), which provides them with an opportunity to acquire viruses from different sources: hosts, food, and associated microflora. These parasites can serve as models for the viral research in other protists helping to better understand viral evolution, ways of transitions between hosts, and molecular mechanisms governing adaptation of viruses to distantly related eukaryotes.

*Note* - While this manuscript was under review, a relevant paper has been published on this topic (Grybchuk et al. 2017, *in press*). The authors demonstrated that diversity of RNA viruses in trypanosomatids is substantially greater than it was envisioned before and revealed that representatives of tombus-like viruses, naranaviruses, leishbunyaviruses, and a novel supergroup (provisionally called “Ostravirus”) can be found in trypanosomatids. Importantly, no relatives of LRV1/2 were documented, keeping these viruses restricted to *Leishmania*. Taken together, these data further justify the need of a broader sampling for trypanosomatid viruses.
