# Supplementary material for: RNA viruses in trypanosomatid parasites: a historical overview
Source: Mem Inst Oswaldo Cruz. 2018 Feb 19;113(4):e170487. doi: 10.1590/0074-02760170487 (PMC5851034; doi:10.1590/0074-02760170487)
Supplement: Supplementary file 1 [file 0074-0276-mioc-113-4-e170487-Suppl01.pdf]

TABLE

Characterised representatives of LRV1 and LRV2. GenBank accession number for RDRP sequence and viral origin are shown

	Host	Name	Accession	Origin
LRV1	*Leishmania guyanensis*	LRV1/Lg2028_3	KY750616	French Guyana
		LRV1/LgLL28	KY750618	French Guyana
		LRV1/Lg2028_1	KY750614	French Guyana
		LRV1/LgXK73	KY750627	French Guyana
		LRV1/LgYE48	KY750628	French Guyana
		LRV1/LgVL91	KY750622	French Guyana
		LRV1/LgWF69_2	KY750625	French Guyana
		LRV1/LgWF69_1	KY750624	French Guyana
		LRV1/Lg2028_2	KY750615	French Guyana
		LRV1/LgXJ93_1	KY750626	French Guyana
		LRV1/LgMJ25	KY750620	French Guyana
		LRV1/Lg2015	KY750613	French Guyana
		LRV1/LgVW21	KY750623	French Guyana
		LRV1/LgYZ58	KY750630	French Guyana
		LRV1/Lg2014	KY750611	French Guyana
		LRV1/LgYR07	KY750629	French Guyana
		LRV1/LgPD46	KY750621	French Guyana
		LRV1/Lg2008	KY750612	French Guyana
		LRV1/LgLF94	KY750608	French Guyana
		LRV1/LgMC71	KY750619	French Guyana
		LRV1/LgXJ93_2	KY750609	French Guyana
		LRV1/LgLF98	KY750617	French Guyana
		LRV1/Lg2001	KY750607	French Guyana
		LRV1/LgyM4147	KX808487	Brazil
		LRV1/MHOM/SR/80/CUMC1	M92355	Surinam
	L. braziliensis	LRV1/LbrLEM2700	KX808483	Bolivia
		LRV1/LbrLEM2780(a)	KX808484	Bolivia
		LRV1/LbrLEM2780(b)	KX808485	Bolivia
		LRV1/LbrLEM3874	KX808486	Bolivia
		LRV1/LbrYA70	KY750610	French Guyana
		LRV1/MHOM/BO/2011/2169	KC862308	Bolivia
LRV2	*L. aethiopica*	LRV2/MHOM/ET/2011/LDS327	KF256265	Ethiopia
		LRV2/MHOM/ET/2011/LDS303	KF256264	Ethiopia
		LRV2/Lae-L494	KF757256	Ethiopia
	L. major	LRV2-1/MHOM/SU73/5-ASKH	U32108	Turkmenistan
	L. infantum	LRV2/IR/2014/HM-2 partial	KP054245	Iran
		LRV2/IR/2014/HM-1 partial	KP054244	Iran

cc BY
